# Healthier Cities through Systems Thinking: Practical Considerations for City Leaders

**DOI:** 10.1007/s11524-022-00655-1

**Published:** 2022-06-28

**Authors:** Damodar Bachani, Sweta Saxena, Muhammad Amri Akbar, Rishav Gupta, Saroj Basnet, Amanda Pomeroy-Stevens

**Affiliations:** 1 Building Healthy Cities Project, John Snow India Private Limited, New Delhi, India; 2 United States Agency for International Development, Bureau for Asia/Technical Services, Washington, DC USA; 3 Social Culture Government Department, Makassar, Indonesia; 4 Indore Smart City Development Limited, Indore, India; 5 City Planning Commission, Kathmandu, Nepal; 6 Building Healthy Cities Project, JSI Research & Training Institute, Inc., Arlington, VA USA

## Commentary

Cities must plan for their future, or risk outgrowing their available resources. Urban populations are becoming denser and more diverse, while at the same time demanding more healthy, livable environments [1]. It is difficult to plan well for inclusive, healthy city growth, in part because urban planners and policymakers must consider the impact of a large number of projects on the health of urban residents. By linking urban planning and health sector goals, planners can better account for the complex inter-connections and dynamics between and across urban services [2–4]. A 2009–2011 Lancet Commission developed five recommendations for how urban planning can improve health outcomes, summarized as [2]: (1) working with a wide range of stakeholders to build political alliances for urban health; (2) focus on urban health inequalities when planning the urban environment; (3) change the urban environment to maintain the urban advantage in health outcomes; (4) do complexity analysis to help policymakers understand the many overlapping relationships affecting urban health outcomes; and (5) experiment locally to create effective actions for urban health.

So how do cities apply these practices? The United States Agency for International Development (USAID)-funded Building Healthy Cities (BHC) project has worked with four Asian cities to test new models of “healthy” urban planning with these recommendations in mind. In Makassar (Indonesia), Indore (India), Da Nang (Vietnam), and Kathmandu (Nepal), BHC used a systems approach to assess and address complex challenges. This allowed BHC and city partners to identify strategies that are immediately relevant to city leadership; make cities healthier and livable; address both short- and long-term goals; and better represent and engage all city residents in feedback loops. Here, leaders from BHC’s partner cities shared their experiences of using this systems approach and tools to guide more efficient and effective planning for healthy urban growth. This commentary may prove useful to peer cities as they consider the practicality of applying systems thinking methodologies to urban planning and development.

### Linking Urban Planning and Health Outcome Goals

BHC’s partner cities were selected because they were fast-growing, Smart Cities^1^ with dynamic contexts. All cities were already active in urban planning but may not have had successes in addressing urban health specifically. BHC chose to engage Smart City mechanisms as they offered greater funding flexibility, opportunities to lead multi-sector collaboration, information and communications technology (ICT) capabilities, prospects for built environment improvements, and some offered an existing focus on citizen engagement. All of these are critical not just for urban planning, but also for improving urban health.

### Smart City Governance Models and the Urban Environment

Each city has a different model for achieving Smart City goals, including urban health. In Makassar, the Regional Planning and Development Agency’s (Bappeda) role is to carry out urban development and oversee all aspects of technical policies. While Bappeda coordinates many projects including Smart City projects, no one city agency manages the funding for Smart City strategies or programs. Bappeda also tracks urban health indicators that measure quality of life and are influenced by the living environment. In Indore, a Special Purpose Vehicle — Indore Smart City Development Limited (ISCDL) — oversees Smart City activities, and is coordinated by the National Smart City Mission [5], ISCDL has a Chief Executive Officer (CEO), who is primarily responsible for implementing Smart City initiatives and achieving specific outcomes (along with a board and oversight structure) [6]. “Smart Health” is one of several categories that ISCDL oversees [7].

In Da Nang, the Department of Information and Communications advises the Da Nang City People’s Committee on implementing policies for building a Smart City, including use of ICT. The Smart City work in Da Nang includes a category for “Smart [health] care” [7]. Smart City activities in Kathmandu are overseen by the Kathmandu Valley Development Authority, but BHC was asked to work specifically on the rollout of the Kathmandu Valley Air Quality Action Plan (KVAP), proposed by the Department of Environment and approved by the national cabinet in February 2020 [8]. While the KVAP is focused on mitigating air pollution (an urban health issue), this multi-sector effort is similar to Smart City initiatives as it aims to address underlying environmental and structural factors contributing to this issue while also strengthening capacity (including ICT and monitoring systems) and increasing citizen awareness and participation.

Partner cities were already making improvements to the urban environment and ICT infrastructure prior to BHC [9–11]. BHC efforts expand on these activities by linking city planning and spending for the urban environment and ICT more explicitly to urban health goals. Systems mapping has helped foster a dialogue between citizens and government regarding the services and physical resources needed to live healthy lives, and how these interconnect to compete with (e.g., easy transport versus green space) or complement each other (e.g., effective waste management and improved air quality).

### Multi-Sectoral Engagement

Muhammad Amri Akbar, Head of the Social Culture and Government Department in Makassar, shared that the government is not solely responsible for developing the city, but that non-governmental organizations, academics, private sector actors, and the community (community-based organizations and citizens) are also responsible. “Urban development needs holistic thematic discussion, integrative coordination, and spatial mapping to identify problems and find solutions to tackle the problems,” noted Amri Akbar. The BHC team in Makassar collaborated with Bappeda to strengthen engagement across stakeholders and sectors through regular meetings of the Healthy City Forum. This forum has provided a space, both online and offline, for discussion on various thematic issues related to urban health. One example Amri Akbar shared relates to sanitation — while this is the jurisdiction of the Public Works Department, its effects impact child stunting, which is under the jurisdiction of the Health Office. In addition, collecting the information needed to improve sanitation requires the involvement of the Communication and Information Department.

In Indore, ISCDL has been using a multi-sectoral approach in the planning and implementation of Smart City projects to ensure effective implementation. Rishav Gupta, CEO of ISCDL, explained that ISCDL ensures smooth coordination with other government departments and developmental agencies. To extend this spirit of collaboration to health-related activities, the BHC team in Indore and ISCDL constituted and convened the Multi-sectoral Smart Health Working Group, which reviews proposed activities related to health and social determinants of health. The group has already generated a collaboration between the Education and Health Departments to re-allocate empty schools to become health centers.

Saroj Basnet, Vice Chairman of the City Planning Commission for Kathmandu Metropolitan City, noted that the recent COVID-19 pandemic has highlighted the need for city managers to adopt a multi-sectoral approach, not only from the perspective of health risks but also for socioeconomic issues as many migrant workers were left stranded, jobless, and without means to return home. In Da Nang, BHC was told by city leaders that multi-sectoral engagement is essential to ensure the goal of “leave no one behind” in building a smart and digitally transformative city.

### Equitable Citizen Engagement and Participatory Planning

In order to increase equity, more voices need to be included during planning processes. However, one common pitfall of participatory planning approaches is that city leaders can become overwhelmed with many requests for new projects and funding. They must judiciously and efficiently prioritize available resources and justify decisions to all stakeholders. The systems maps provide a transparent tool to facilitate citizen engagement and inform this type of decision-making. As Basnet explained, a “systems approach can be a very useful method to engage citizens and other stakeholders in urban planning and development process. The objective of a systems approach is to provide a bigger picture of [the] issue on hand and to recommend interventions.”

In the past, the Makassar administration used various approaches to identify and prioritize programs for implementation. They have existing bottom-up planning approaches called Musrenbang, starting from the sub-district level, which must align with top-down directives, indicators, and outcome goals provided by the national level [12]. After Bappeda partnered with BHC, the city began using the systems map tools for prioritization during Musrenbang. “We can use the systems approach and integrate it with the approaches that we have been using to obtain most effective, efficient, and comprehensive results,” said Amri Akbar. Overall, Amri Akbar felt that the approach is rational since it is scientific and evidence-based, and the map facilitation workshop was helpful in identifying problems and connecting patterns of cause and effects.

In Indore, city planners conducted an extensive citizen engagement campaign when developing the Smart City Master Plan and discussed it with all relevant stakeholders before finalization. “We have ensured inclusion of demands of citizens, especially vulnerable population, in our work,” said Gupta. He added that the participatory aspect of the systems approach is valuable because “citizens are the main stakeholders… [integral] to the whole process as we are planning for them and their needs.” If the participatory mapping technique is led and co-created by citizens and local leaders, then the map provides a truly local perspective that leads to localized solutions.

### Caveats and Challenges

All three city administrators identified some challenges in introducing a systems approach. Systems thinking is a complex approach for urban planning and requires building the capacity of various stakeholders to exercise a new mindset of looking holistically at city systems. Capacity for data use and visualization is also required. Moreover, system maps should be simple and clear so that the intended meanings are not lost in translation when developing action plans. The systems approach should be used as a time- and resource-saving process that can easily be implemented by any government department.

Implementing a systems approach requires a long discussion process, and synergy between various stakeholders is required to produce a useful program design. Transforming potential solutions into an action plan was perceived as a challenge due to a lack of ownership, particularly when responsibility for an action spans more than one sector.

### Planning for Complexity

Basnet mentioned that tools such as mind maps, logical frameworks, and BHC’s systems maps can break down complex urban issues into simpler components. Gupta found the systems approach to be an innovative method for demonstrating the reality of the current system, its strengths, its weaknesses, and areas that need attention. “It is a complex approach for urban planning, but certainly this is a new way of looking and analyzing holistically a system. We will use this as a tool for planning health projects in the future,” said Gupta.

Gupta said that it can easily be used by any government department when a rapid, planning process may be required — this is important to consider in times of shock while planning for the resiliency of urban systems. Basnet suggested though that the underlying assumptions for the systems maps must be clearly stated. The systems approach is meant to simplify the otherwise complicated connections between interventions or actions.

BHC is now synthesizing evidence and identifying what other cities might be able to learn from these healthy urban planning experiences. Weighed against the recommendations of the Lancet Commission, the BHC systems approach, combined with the strengths of Smart City governance models, covers the key points relating to addressing complexity and strengthening multi-sectoral engagement, equity, and localization of solutions for urban health. The successful future growth of cities will depend on an emerging cadre of human resources trained to effectively navigate the complexity of urban settings and achieve improved health outcomes. Our co-authors in city leadership have identified some of the benefits and challenges of applying systems thinking to healthy urban planning. These considerations can help refine this approach for greater practicality, simplicity, usability, and relevance to urban planning mechanisms in Asia and beyond.

## Footnotes

1 Smart Cities are defined differently in each country, but generally include efforts meant to improve both information and communications technology as well as the built environment in that city.
